# Webster's Unabridged Dictionary

**Published:** 1874-01

**Authors:** 


*Webster's Unabridged Dictionary,*—Ten thousand *words and meanings not in other Dictionaries.* Three thousand Engravings. 1840 pages quarto. Price $12.00.

In the original preparation of Webster's Dictionary, Dr. Tully, a physician of eminence and great learning, took a prominent part. In the last revision, 1864, the medical department was carefully revised, and, as stated in the Preface, "In Physiology and Medical Science, Professor R. Cresson Stiles, M. D., of the Medical School of Yale College, has furnished many carefully considered definitions and emendations," whilst in Botany, Chemistry, and kindred Natural Sciences, a thorough revision, by the most competent scholars, took place.

What volume, next to purely professional books, (and this is hardly less, medically, than a professional one,) of greater and more constant usefulness to the medical student and practitioner than Webster's Unabridged Dictionary?

"Excels all others in defining scientific terms."—*President Hitchcock.*

☞ A National Standard. The authority in the Government Printing Office at Washington, and supplied by the Government to every pupil at West Point.

Webster's Dictionary is the Standard authority for printing in the Government Printing Office, and has been for the last four years.—A. M. Clapp, *Congressional Printer.*

☞ Warmly recommended by Bancroft, Prescott, Motley, Geo. P. Marsh, Halleck, Whittier, Willis, Saxe, Elihu Burritt, Daniel Webster, Rufus Choate, and the best American and European scholars.

A necessity for every intelligent family, student, teacher, and professional man. What Library is complete without the best English Dictionary? Also Webster's National Pictorial Dictionary. 1040 pages octavo. 600 Engravings. Price $5.00. Published by G. & C. Merriam, Springfield, Mass.

